# UNVEILING LONGEVITY INSIGHTS: AFRICAN AMERICAN OLDER ADULTS IN THE HEALTH AND RETIREMENT STUDY

**DOI:** 10.1093/geroni/igae098.0381

**Published:** 2024-12-31

**Authors:** Meneka Johnson Nicholson, Peter Martin

**Affiliations:** Rural Health Medical Program, Inc., Selma, Alabama, United States; Iowa State University, Ames, Iowa, United States

## Abstract

Although there is considerable longevity literature, few studies have focused on African American older adults. The current study assessed different domains of longevity predictors for African American participants in the Health and Retirement Study. A total of 723 African Americans (Mage = 68.2) who participated in wave 2 of the HRS were included in this study. Family longevity, individual and social resources, health behaviors and health were used to predict their age at death (Mage = 79.5). A blockwise multiple regression was used for the analyses. Results indicate that father’s and mother’s age at death did not predict longevity. Education and number of children were significant predictors when added as a second block (β = -.24, p <.05 and β =-.13, p <.05, respectively). Of the next block, body-mass index and smoking (β = -.10, p <.05 and β =-.26, p <.05, respectively), but not alcohol intake, added significantly to explaining longevity. Four specific health markers (i.e., self-rated health, high blood pressure, diabetes, and multimorbidity, β = -.08, p <.05, β =.30, p <.05, β = -.26, p <.05, β =-.29, p <.05, respectively) all added significantly to the explanation of longevity. The two final blocks (ADL functioning and level of depression), did not add significantly to the equation. On the whole, the model explained 35 percent of the variance. The results are important for African Americans, policymakers and practitioners as they can help identify risk and resilience factors of longevity.

